# How to demonstrate the clinical implication of prophylactic implantable cardioverter–defibrillator in patients with advanced light chain amyloidosis

**DOI:** 10.1002/joa3.70099

**Published:** 2025-05-26

**Authors:** Naoya Kataoka, Teruhiko Imamura

**Affiliations:** ^1^ Second Department of Internal Medicine University of Toyama Toyama Japan

**Keywords:** atrial fibrillation, cardiac amyloidosis, heart failure, ventricular tachyarrhythmia

To Editor,

The indication for implantable cardioverter–defibrillator (ICD) implantation in patients with cardiac amyloidosis remains a matter of ongoing debate. Although the incidence of sudden cardiac death in this population is reported to be approximately 10%–30%, the majority of these events appear to result from pulseless electrical activity, which is unresponsive to defibrillation. The authors observed that ventricular arrhythmias were frequently present in patients with advanced light chain (AL) amyloidosis, and that ICD therapy was effective in terminating sustained ventricular arrhythmias in this subgroup.^1^ However, several concerns warrant consideration.

The current study included only 10 patients with AL amyloidosis, most of whom appeared to exhibit heart failure with preserved ejection fraction.^1^ Generally, ICD implantation is not recommended for patients with preserved left ventricular ejection fraction, except for those with cardiac sarcoidosis.^2^ Do the authors advocate ICD implantation in all patients with AL amyloidosis, irrespective of left ventricular function? The cost‐effectiveness of such an approach merits careful evaluation.

Notably, 40% of the cohort received single‐chamber ICDs.^1^ Given that patients with cardiac amyloidosis frequently develop supraventricular arrhythmias, such as atrial fibrillation, the potential for inappropriate ICD shocks cannot be overlooked.^3^ It remains unclear whether inappropriate shocks occurred in this cohort.

Recent studies have highlighted the potential role of catheter ablation in managing ventricular arrhythmias in patients with cardiac amyloidosis.^4^ In light of the authors' finding that appropriate shocks were commonly delivered,^1^ prophylactic catheter ablation may represent a viable alternative strategy. The influence of AL‐directed chemotherapy on the incidence and burden of ventricular arrhythmias also deserves further investigation.

## CONFLICT OF INTEREST STATEMENT

Authors declare no conflict of interests for this article.

## Data Availability

The manuscript does not include any original data.
